# Late-stage diagnosis: The driving force behind high breast cancer mortality in Ethiopia: A systematic review and meta-analysis

**DOI:** 10.1371/journal.pone.0307283

**Published:** 2024-07-19

**Authors:** Habtamu Geremew, Eyasu Bamlaku Golla, Mulat Belay Simegn, Alegntaw Abate, Mohammed Ahmed Ali, Hawi Kumbi, Smegnew Gichew Wondie, Misganaw Asmamaw Mengstie, Werkneh Melkie Tilahun

**Affiliations:** 1 College of Health Science, Oda Bultum University, Chiro, Ethiopia; 2 Department of Public Health, College of Medicine and Health Science, Debre Markos University, Debre Markos, Ethiopia; 3 Department of Medical Laboratory Science, College of Health Science, Oda Bultum University, Chiro, Ethiopia; 4 Department of Midwifery, College of Health Science, Oda Bultum University, Chiro, Ethiopia; 5 Department of Laboratory, Adama Hospital Medical College, Adama, Ethiopia; 6 Department of Human Nutrition, College of Medicine and Health Science, Mizan Tepi University, Mizan Aman, Ethiopia; 7 Department of Biochemistry, College of Medicine and Health Sciences, Debre Tabor University, Debre Tabor, Ethiopia; Centro per lo Studio e la Prevenzione Oncologica, ITALY

## Abstract

**Introduction:**

Breast cancer continues to be the most common malignancy and the leading cause of cancer-related deaths in Ethiopia. The poor prognosis and high mortality rate of breast cancer patients in the country are largely caused by late-stage diagnosis. Hence, understanding the epidemiology of late-stage diagnosis is essential to address this important problem. However, previous reports in Ethiopia indicated inconsistent findings. Therefore, this literature review was conducted to generate dependable evidence by summarizing the prevalence and determinants of late-stage diagnosis among breast cancer patients in Ethiopia.

**Methods:**

Pertinent articles were retrieved by systematically searching on major electronic databases and gray literature. Data were extracted into an Excel spreadsheet and analyzed using the STATA 17 statistical software. The pooled estimates were summarized using the random effect meta-analysis model. Heterogeneity and small study effect were evaluated using the I^2^ statistics and Egger’s regression test in conjunction with the funnel plot, respectively. Meta-regression, sub-group analysis, and sensitivity analysis were also employed. Protocol registration number: CRD42024496237.

**Results:**

The pooled prevalence of late-stage diagnosis after combining reports of 24 studies with 8,677 participants was 65.85 (95% CI: 58.38, 73.32). Residence (adjusted OR: 1.92; 95% CI: 1.45, 2.53), patient delay at their first presentation (adjusted OR: 2.65; 95% CI: 1.56, 4.49), traditional medicine use (adjusted OR: 2.54; 95% CI: 1.89, 3.41), and breast self-examination practice (adjusted OR: 0.28; 95% CI: 0.09, 0.88) were significant determinants of late-stage diagnosis.

**Conclusion:**

Two-thirds of breast cancer patients in Ethiopia were diagnosed at an advanced stage. Residence, delay in the first presentation, traditional medicine use, and breast self-examination practice were significantly associated with late-stage diagnosis. Public education about breast cancer and its early detection techniques is crucial to reduce mortality and improve the survival of patients. Besides, improving access to cancer screening services is useful to tackle the disease at its curable stages.

## Introduction

Breast cancer is the world’s most common cancer in women with an estimated 2.3 million incident cases and 685,000 deaths in 2020 alone [1,2]. During the same year, the Sub-Saharan region contributed the highest incidence and mortality of breast cancer with 1,109,209 cases and 25,626 deaths, which makes it one of the major public health issues in the area [2]. In Ethiopia, breast cancer is the leading malignancy, accounting for about one-third of all cancer cases among women [3]. Annually, an estimated 16,133 new breast cancer cases and 9061 deaths occur in the country [4].

The World Health Organization (WHO) inaugurated the Global Breast Cancer Initiative (GBCI) aiming to reduce global breast cancer mortality by 2.5% per year and prevent 2.5 million breast cancer deaths worldwide between 2020 and 2040 [5]. To achieve these goals, WHO urged to have early diagnosis and treatment of breast cancer, but recent data shows that most breast cancer patients are diagnosed at a late stage [6,7].

Late-stage diagnosis has multifaceted consequences that affect the patients, their families and the community at large [8,9]. Stage at diagnosis is an important predictor of survival; late-stage diagnosis increases the mortality risk by more than 82% [10,11]. Patients diagnosed at an advanced stage also had a higher risk of recurrence and poor prognosis [12,13]. Besides, late-stage diagnosis is also associated with poor quality of life, increased healthcare costs and treatment-related problems [8,14].

According to different studies, late-stage breast cancer diagnosis is associated with a low level of awareness about the disease and its screening, poor facilities for accurate and timely diagnosis and treatment, negative symptom interpretation, fear, consideration of traditional healing, and lack of trust and poor access to healthcare [9,15–17].

In Ethiopia, cancer care is pioneered by the only oncology and cancer referral center (Black Lion Specialized Hospital, which is found in the national capital (Addis Ababa) and is providing services for the majority of cancer cases from all over the country [18]. However, recently, Hospitals in different regions of the country are inaugurating cancer care, and this may enhance access; hence, promoting early diagnosis. Previously, various studies have been conducted and found a widely varying prevalence of late-stage breast cancer diagnosis and identified different factors associated with late presentation in different parts of the country [7,19–22]. However, summarized national evidence about the prevalence and determents of late-stage diagnosis among breast cancer patients is lacking. Thus, in this analysis, we aimed to assess the pooled prevalence and determinants of late-stage diagnosis among breast cancer patients in Ethiopia.

## Methods and materials

### Study protocol registration and reporting

This study was registered in the International Prospective Register of Systematic Reviews (PROSPERO) database with protocol number: CRD42024496237. The findings of this review are reported in accordance with the Preferred Reporting Items for Systematic Reviews and Meta-Analyses (PRISMA) statement [23], (S1 Checklist).

### Information source and search strategy

Relevant articles were retrieved by conducting a comprehensive web-based search on PubMed, Scopus, EMBASE, African Journals Online (AJOL), Hinari, Epistemonikos, CINAHL, and Cochrane Library. Additionally, other gray literature sources like Google Scholar and online repositories of Ethiopian universities were examined. The reference lists of pertinent studies were also scrutinized to identify additional reports. Two members of the review team (HG and MBS) searched the databases independently from December 1 to 26, 2023. The database search was conducted using the following keywords: “delayed diagnosis” OR “late-stage diagnosis” OR “advanced stage diagnosis” OR “delayed presentation” OR “advanced stage presentation” OR “end-stage diagnosis” OR “stage at diagnosis” OR stage AND “breast cancer” OR “mammary cancer” OR “breast tumor” OR “breast neoplasm” OR “breast carcinoma” OR “breast malignancy” AND Ethiopia.

### Eligibility criteria

The eligibility criteria for this review were based on CoCoPop (condition, context, and population) mnemonic [24]. Correspondingly, all primary studies published in the English language and reported the number and/or prevalence and/or associated factors of late-stage diagnosis among breast cancer patients in Ethiopia and fulfill the following criteria (Table 1). were included in this review.

**Table 1:** Inclusion and exclusion criteria.

Study characteristics	Inclusion criteria	Exclusion criteria
Design	All observational studies (Cohort, Case-control and Cross-sectional)	Clinical trials, qualitative studies, editorial letters, case report/series.
Population	Breast cancer patients	Patients with malignancies of other body parts
Condition	Late-stage diagnosis of breast cancer	Unclear stage at diagnosis
Context	Studies conducted in Ethiopia	Studies not from Ethiopia
Language	Published in English	Published in other languages

All studies that fulfilled the aforementioned criteria and clearly described that staging was conducted at the time of diagnosis were considered in this analysis. No consideration was given to the time period of publication. Nevertheless, duplicate studies, abstracts without full text and qualitative studies without outcome of interest were excluded from this analysis. Exclusive electronic request messages were sent to authors for full articles not available online or to clarify data.

## Outcome measurement

This systematic review had two major outcomes. The primary outcome was the prevalence of late-stage diagnosis among breast cancer patients in Ethiopia. The secondary outcome of this analysis was to identify factors associated with late-stage diagnosis of breast cancer patients in Ethiopia.

Late-stage or advanced-stage breast cancer: breast cancer diagnosed at stage Ⅲ or stage Ⅳ, irrespective of the staging being clinical or pathological [25,26].

Early-stage breast cancer: breast cancer diagnosed at stage Ⅰ or stage Ⅱ [6,26].

Patient presentation delay: is the time from the patient’s first detection of the first symptom/breast abnormality until their first healthcare facility visit and it was categorized as ≥3 months (long presentation delay) and <3 months (short delay) [20,27].

### Study identification, quality assessment, and data extraction

Records identified through the electronic database search were exported to a reference management software (Endnote version X7.2), where duplicate entries were eliminated. The remaining articles were then assessed by title and abstract. For articles found to be pertinent by title and abstract, a full-text review against the specified inclusion/exclusion criteria was conducted to identify potential articles to be included in this review.

To evaluate the quality of the studies, we utilized the Joanna Brigg’s Institute (JBI) quality assessment checklist [28]. Two authors of the review (HG and EBG) independently evaluated the quality of each study and inconsistencies were resolved by involving a third author (MBS).

Records of primary studies were abstracted into a standard data extraction, Excel spreadsheet form that was developed by considering the JBI guide for data extraction and synthesis [29]. Two authors of the review extracted the data independently and inconsistencies were resolved by discussion. Extracted data include last name of the first author and year of publication, study area, sample size and number and/or prevalence of late-stage diagnosis. Besides, data about predictors of late-stage diagnosis were also abstracted in the form of a two-by-two table.

### Data management and analysis

The necessary data were extracted into Excel spreadsheet and then exported to the STATA version 17 software for further statistical analysis. The general features of primary studies are described in tables. The prevalence and its respective standard error in each primary study were considered to compute the pooled prevalence estimates. The pooled odds ratio estimates were computed after extracting the necessary data into a two-by-two table format. The random effects model was employed to estimate the pooled effect size, and estimates were depicted by forest plots. Heterogeneity among included reports was evaluated using the I^2^ statistics and it was considered as high, moderate, or low when I^2^ test statistics results were 75%, 50%, and 25% respectively [30]. Subgroup analysis was conducted to further investigate the potential source of heterogeneity considering the study area, study design and data collection methods of primary studies. Funnel plot and Eggers regression test were used to assess publication bias, and Eggers regression test with a p-value less than 0.05 was considered to indicate the presence of publication bias [31]. The observed substantial heterogeneity between included studies was also further explored by subgroup analysis. Besides, the impact of each study on the overall estimate was investigated using a leave-one-out sensitivity analysis.

### Ethics approval and consent to participants

Not applicable because no primary data were collected.

## Results

### Identification of records

**Fig 1:**
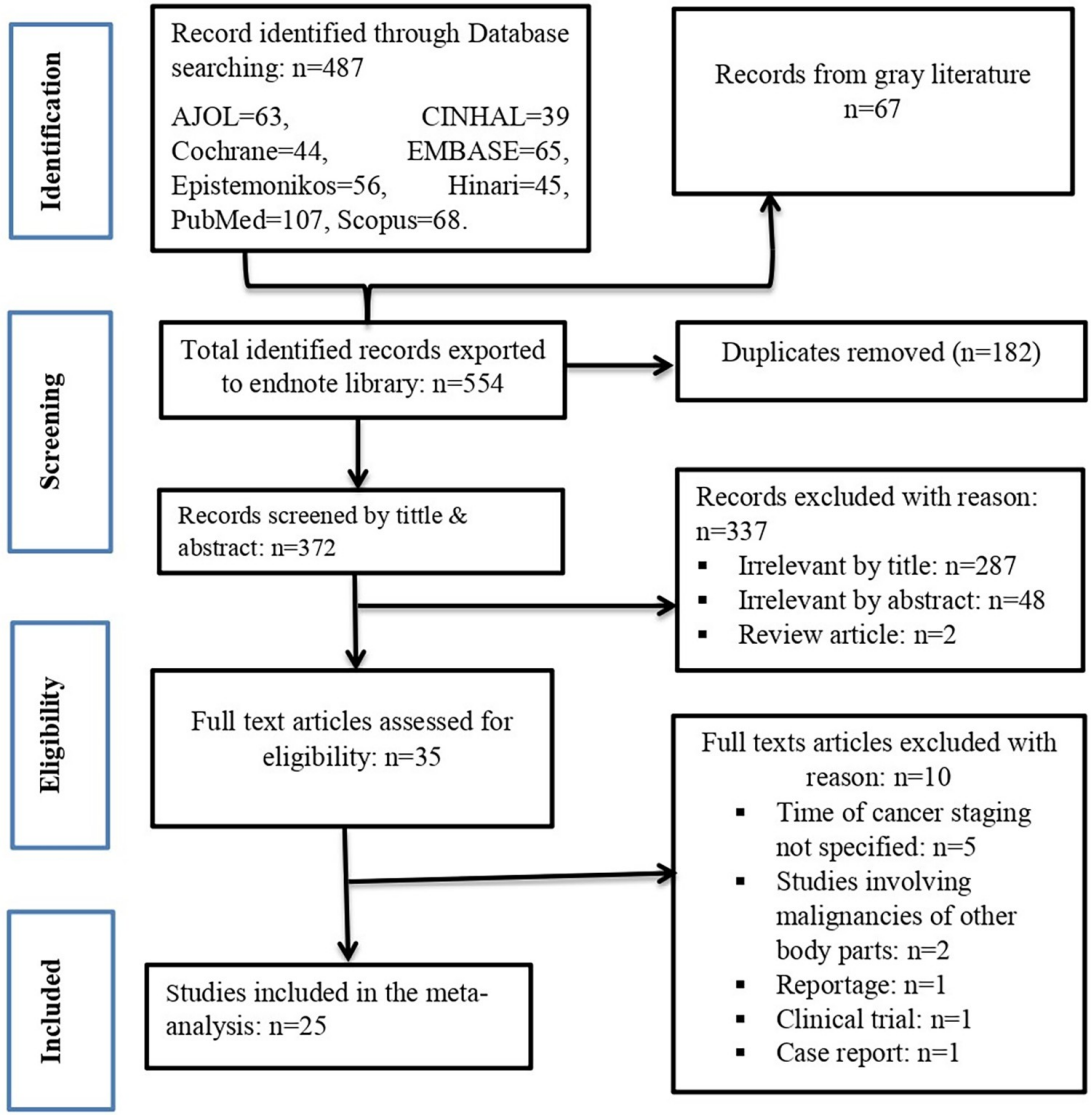
PRISMA flow chart for articles screened and included.

A total of 554 primary studies were retrieved through the combined electronic literature search. Of these, 182 records were removed due to duplication. Assessment of the title and abstract of the remaining records eliminated 337 studies. Then, the full texts of 35 records were evaluated against the eligibility criteria, and 25 were included in the meta-analysis (**Fig 1**).

### Overview of included studies

In this review, a total of 25 studies with 8,986 participants were considered. The sample size of included studies was substantially variable ranging from 128 [32] to 787 [33]. Regarding the study design of primary studies, 16 studies were cross-sectional [7,20–22,25,27,32–41], 8 were cohort [10,11,19,42–46], and 1 study employed case-control study design and it was considered only for the determination of associated factors [47]. Most (20) studies included women participants only [7,10,11,19,20,22,25,27,33–36,39,40,42–46], while 5 studies involved both women and men [21,32,37,38,41]. According to the findings of primary studies, the prevalence of late-stage diagnosis varies between 15.2% [42] and 86.3% [34] (**Table 2**).

**Table 2:** Characteristics of included studies.

First author & year of publication	Study area	Study design	Data collection method	Total participants	Age in years	Prevalence of LSD*	Risk of bias
Gebremariam et al, 2020 [25]	Addis Ababa	Cross-sectional	Interview and record review	406	44.4**	64.3%	Low risk
Shewarega et al, 2023 [7]	Addis Ababa	Cross-sectional	Interview and record review	269	44**	66.9%	Low risk
Tesfaw et al, 2021, A [20]	Regions	Cross-sectional	Interview and record review	371	40^#^	71.2%	Low risk
Tesfaw et al, 2021, B [21]	Regions	Cross-sectional	Record review	426	42.78**	72.5%	Low risk
Yoseph et al, 2021 [34]	Regions	Cross-sectional	Interview and record review	255	NR	86.3%	Low risk
Hassen et al, 2019 [39]	Addis Ababa	Cross-sectional	Interview and record review	403	44**	60.1%	Low risk
Dagne et al, 2019 [40]	Addis Ababa	Cross-sectional	Record review	303	42.1^#^	68.0%	Low risk
Ersumo et al, 2018 [41]	Addis Ababa	Cross-sectional	Record review	347	46^#^	54.2%	Low risk
Gebretsadik et al, 2021 [37]	Regions	Cross-sectional	Record review	559	38^#^	66.5%	Low risk
Hassen et al, 2021 [22]	Regions	Cross-sectional	Interview and record review	204	44.1**	66.2%	Low risk
Kantelhardt et al, 2014 [33]	Addis Ababa	Cross-sectional	Record review	787	NR	76.1%	Low risk
Koboto et al, 2020 [38]	Regions	Cross-sectional	Interview and record review	259	44.89**	79.5%	Low risk
Legese et al, 2021 [36]	Addis Ababa	Cross-sectional	Interview and record review	375	40^#^	64.8%	Low risk
Muhammed et al, 2022 [27]	Regions	Cross-sectional	Interview and record review	150	37.4**	66.0%	Low risk
LM Tesfaw et al, 2020 [32]	Regions	Cross-sectional	Interview and record review	128	NR	85.2%	Low risk
Teshome et al, 2021 [35]	Addis Ababa	Cross-sectional	Interview and record review	188	≥ 18	50.5%	Low risk
Areri et al, 2019 [46]	Addis Ababa	Cohort	Record review	627	42.61**	69.9%	Low risk
Feleke et al, 2022 [45]	Regions	Cohort	Record review	322	43.8**	54.7%	Low risk
Gebremariam et al, 2023 [19]	Addis Ababa	Cohort	Interview and record review	404	44.4**	64.1%	Low risk
Hagos et al, 2023 [44]	Regions	Cohort	Record review	146	48.32**	56.8%	Low risk
Marine et al, 2023 [42]	Regions	Cohort	Record review	552	> 15	15.2%	Low risk
Misganaw et al, 2023 [11]	Regions	Cohort	Record review	412	45**	72.6%	Low risk
Shita et al, 2023 [43]	Regions	Cohort	Interview and record review	302	39^#^	83.4%	Low risk
Truneh et al, 2021 [10]	Regions	Cohort	Interview and record review	482	61^#^	65.1%	Low risk
W/Michael et al, 2018 [47]	Regions	Case-control	Interview and record review	309	43**	NR	Low risk

**Key**; LSD*: Late stage diagnosis, NR: Not reported

**: Mean

^#^: Median.

### Prevalence of late-stage diagnosis

**Fig 2:**
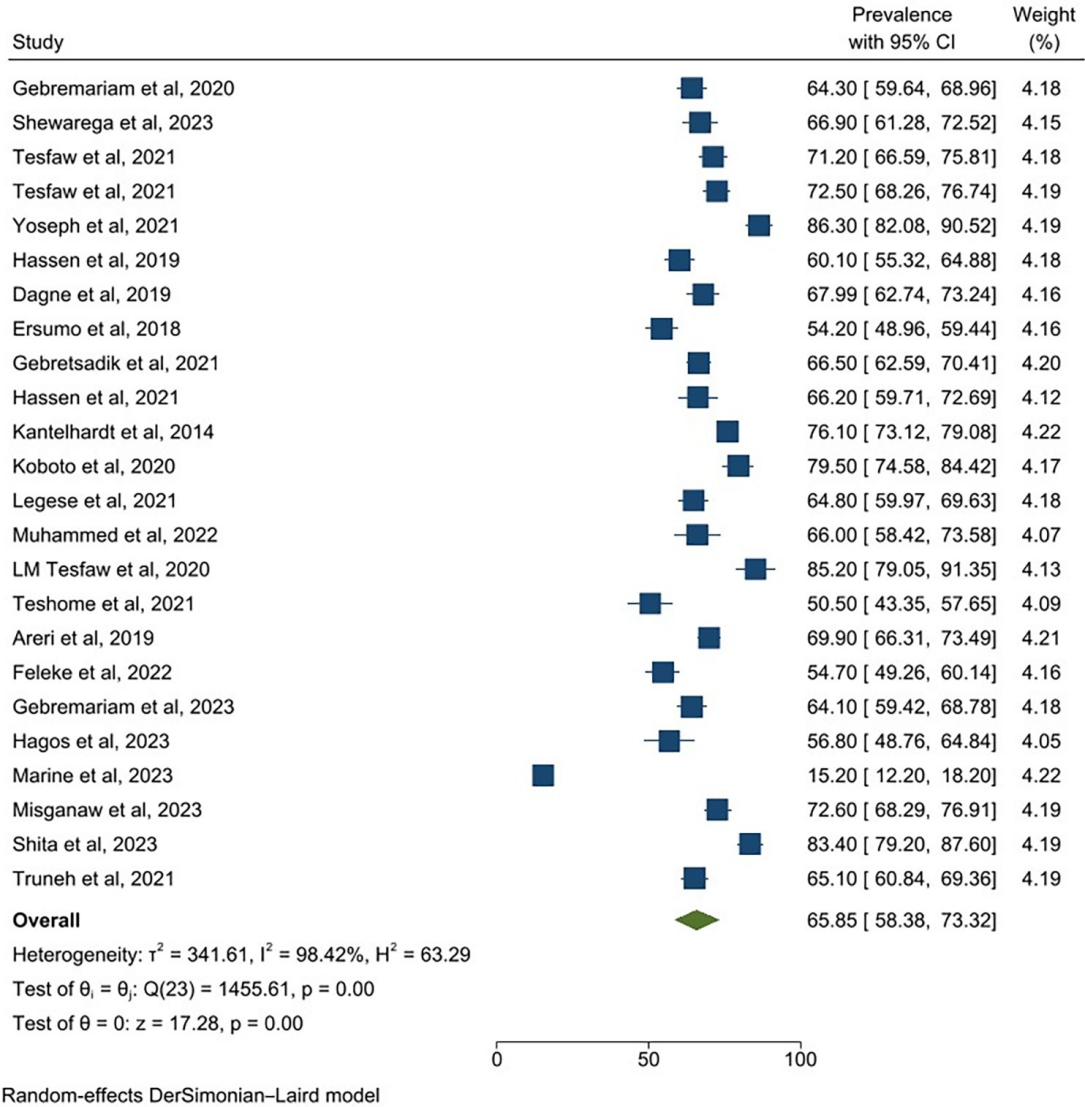
Forest plot for prevalence of late-stage diagnosis among breast cancer patients in Ethiopia.

After combining the findings of 24 studies with a total of 8,677 participants, the pooled prevalence of late-stage diagnosis among breast cancer patients in Ethiopia was 65.85 (95% CI: 58.38, 73.32) (**Fig 2**).

### Heterogeneity, publication bias and sensitivity analysis

A substantial level of heterogeneity (I^2^ = 98.42%) was observed between included studies; for that, the pooled prevalence estimate was computed using the random effect model (DerSimonian-Laird method), in order to take this significant heterogeneity into account. Subgroup analysis was also done based on study area, study design and method of data collection, yet, no significant variation was detected. Besides, the random effect meta-regression was performed to further explore the source of heterogeneity by considering sample size and publication year as covariates. Consequently, the results indicated that sample size and year of publication had no effect on heterogeneity between studies (**Table 3**).

**Table 3:** Meta-regression analysis of factors affecting between-study heterogeneity.

Source of heterogeneity	Coefficient	Standard error	t	P > |t|	95% Confidence Interval
Sample size	-0.0296106	0.0242871	-1.22	0.236	-0.0801184, 0.0208972
Publication year	-2.635856	1.88439	-1.40	0.176	-6.55466, 1.282948
Constant	5403.1	3812.081	1.42	0.171	-2524.556, 13330.76

**Fig 3:**
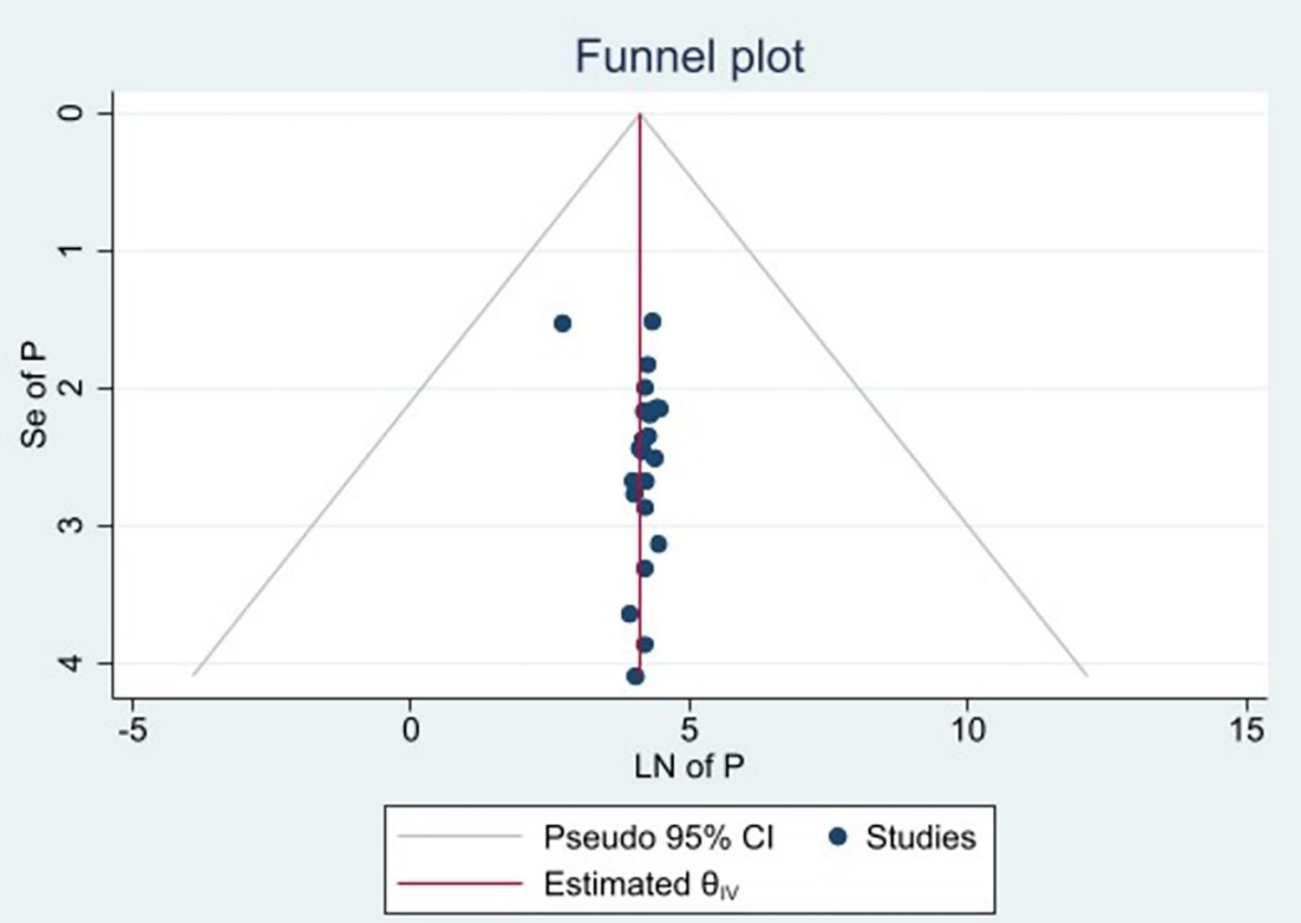
Funnel plot, evaluating existence of publication bias for prevalence of late-stage diagnosis among breast cancer patients in Ethiopia.

**Fig 4:**
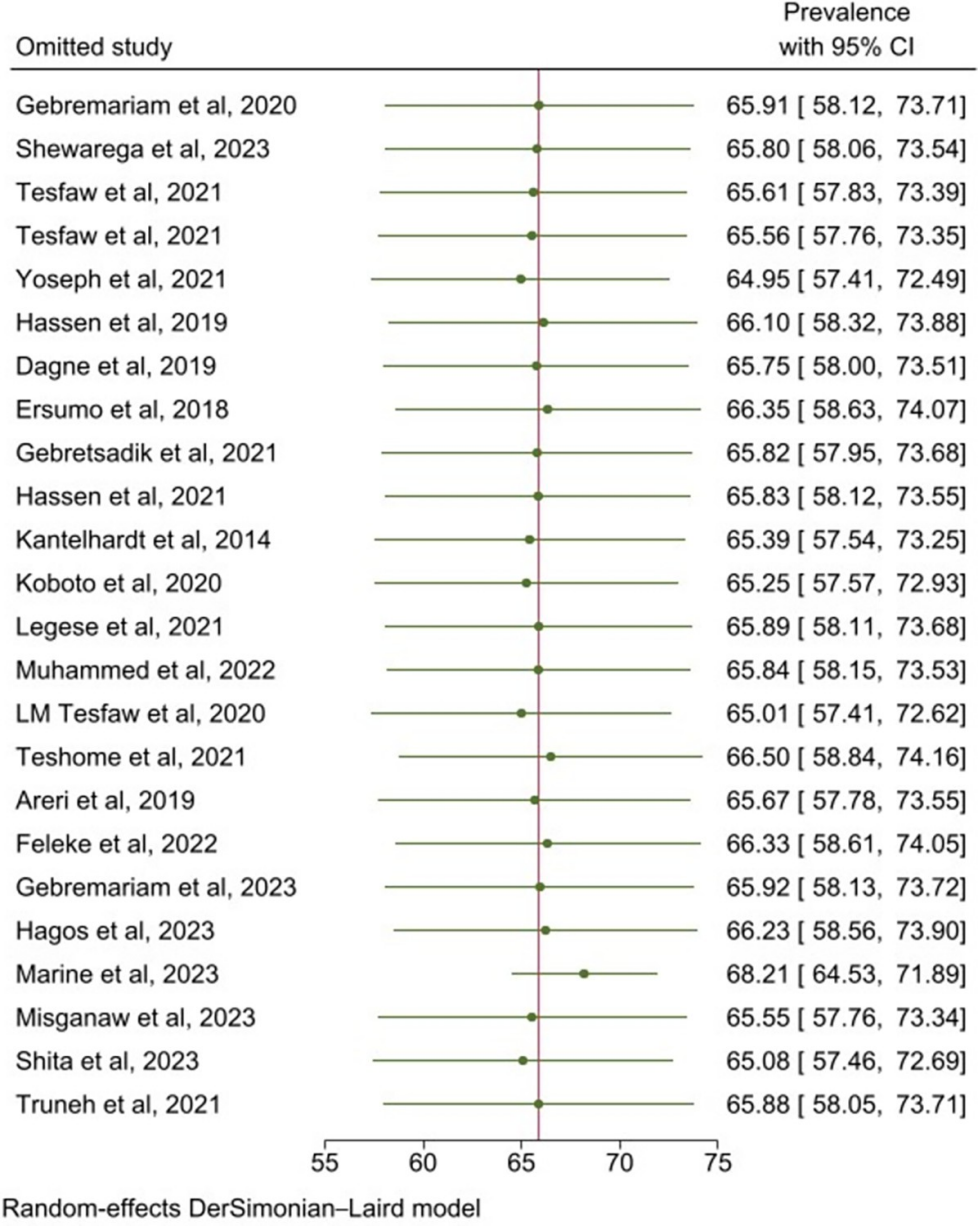
Sensitivity analysis for prevalence of late-stage diagnosis among breast cancer patients in Ethiopia.

Publication bias was evaluated using a funnel plot and Egger’s regression test. As a result, findings of both the funnel plot (**Fig 3**). and Egger’s regression test (p  =  0.95) indicated no evidence of publication bias. Furthermore, the influence of each study on the overall prevalence estimate was investigated using a leave-one-out sensitivity analysis. As a result, the pooled estimates of late-stage diagnosis among breast cancer patients in Ethiopia was steady and reliable when analyzed by removing one study at a time (**Fig 4**).

### Determinants of late-stage diagnosis

Data about 7 variables (educational status, marital status, residence, patient delay, utilization of traditional medicine, family history of breast cancer, and breast self-examination) were extracted into an Excel spreadsheet in the form of a two-by-two table and analyzed separately. Consequently, residence, patient delay, traditional medicine utilization and breast self-examination were found to have a statistically significant association with late-stage diagnosis of breast cancer. In this regard, the risk of late-stage diagnosis was 92% higher among rural residents when compared to urban dwellers (adjusted OR: 1.92; 95% CI: 1.45, 2.53). The odds of late-stage diagnosis were 2.65 times more likely among breast cancer patients who had experienced patient delay during their first presentation as compared to their counterparts (adjusted OR: 2.65; 95% CI: 1.56, 4.49). Similarly, patients who had utilized traditional medicine had a 2.54 times higher risk of late-stage diagnosis relative to their counterparts (adjusted OR: 2.54; 95% CI: 1.89, 3.41). The findings of our analysis also indicated that practicing breast self-examination reduces the risk of late-stage diagnosis by 72% (adjusted OR: 0.28; 95% CI: 0.09, 0.88) (**Table 4**). Conversely, pooling the effect size of some variables deemed meaningless because of the difference in categorization across primary studies. For instance, five studies explored the association between age and late stage diagnosis; however, the effect size was not pooled because all of the studies categorized age inconsistently.

**Table 4:** Summary estimate of OR for factors associated with late-stage diagnosis of breast cancer patients in Ethiopia.

Variables	Exposed	Comparator	Included studies	Total participants	OR (95% CI)	I^2^
Residence	Rural	Urban	4	1204	1.92 (1.45, 2.53)	0.0%
Patient delay	Delayed	Not delayed	4	1353	2.65 (1.56, 4.49)	75.4%
Traditional medicine	Yes	No	3	1032	2.54 (1.89, 3.41)	0.0%
BSE*	Yes	No	2	777	0.28 (0.09, 0.88)	86.9%
Family history	Yes	No	3	936	0.66 (0.43, 1.00)	0.0%

**Abbreviation:** BSE*: Breast Self Examination.

## Discussion

Breast cancer continued to be the most common malignancy and the leading cause of cancer-related deaths in Ethiopia [2]. Late-stage diagnosis significantly contributes to the poor prognosis and high mortality of breast cancer patients [43,48]. In Ethiopia, evidence about the epidemiology of late-stage diagnosis among breast cancer patients is not conclusive. Therefore, we aimed to determine the pooled prevalence and determinants of late-stage diagnosis among breast cancer patients in Ethiopia.

In this study, the pooled prevalence of late-stage diagnosis among breast cancer patients in Ethiopia was 65.85 (95% CI: 58.38, 73.32). This finding is in line with another meta-analysis that summarized the results of studies conducted in Africa, 67% [49]. However, our estimate was higher than a previous meta-analysis conducted in Latin America and the Caribbean, 40.76% [9]. This might be due to the marked difference in socio-economic status and healthcare services between the nations denoted in the primary studies [50]. On the other hand, our prevalence estimate was lower than the finding of an earlier review that summarized the results of studies conducted in 17 sub-Saharan African countries, 77% [6]. The possible explanation for this variation could be due to the difference in the study period of included studies; where, our analysis involved breast cancer patients diagnosed between 2005 and 2022 [33,42], while the sub-Saharan African analysis included breast cancer patients diagnosed between 1957 and 2012 [51,52]; and it is evidenced elsewhere that measures that reduce late-stage diagnosis like breast cancer screening had evolved over time [53]. For example, the recent expansion of cancer care outside the national capital and the inauguration of the national cancer control strategy might contribute to this end [54].

The current review also identified determinants of late-stage diagnosis. Residence, patient delay, traditional medicine use and breast self-examination were found to have a statistically significant association with late-stage diagnosis. The risk of late-stage diagnosis was 92% higher among rural residents when compared to urban dwellers. This association was also detected by previous studies [55,56], and could be due to the lack of awareness and practice of early screening methods among rural dwellers [57,58]. Poor access to preventive cancer care among rural residents could also be another elucidation for this variation [59].

In line with another study [60], this review indicated that breast cancer patients who had experienced patient delay for their first presentation were 2.65 times more likely to be diagnosed at an advanced stage than their counterparts. This might be due to the poor knowledge about signs and symptoms of breast cancer among delayed patients, thereby inhibiting timely consultation and leading to advanced disease stage [17]. Similarly, patients who had utilized traditional medicine before diagnostic confirmation had a 2.54 times higher risk of late-stage diagnosis relative to their counterparts. The plausible explanation for this might be due to the progression of the disease with the lapses of time while receiving traditional medicine [16].

The present study showed that practicing breast self-examination reduces the risk of late-stage diagnosis by 72%. This finding is supported by a previous study conducted in Togo [15]. This might be because regular breast self-examination helps women to understand how their breast commonly feels, which in turn helps them to early notice changes in their breasts, and make them consult clinicians [61]. The finding underlines the importance of regular breast self-examination in the early detection of breast cancer, particularly in resource-constrained settings like Ethiopia [62]. Therefore, strengthening advocacy and integration of the breast self-examination education with outreach healthcare activities might help to reduce late stage diagnosis of patients.

## Limitations of the review

Interpretation of the findings of this meta-analysis should be done with due consideration of the substantial heterogeneity between included studies. Studies that were published only in the English language were taken into account. Besides, variations in categorization between primary studies precluded the inclusion of some variables.

## Conclusion

Two-thirds of breast cancer patients in Ethiopia were diagnosed at an advanced stage. Residence, delay in the first presentation, traditional medicine use and breast self-examination practice were significantly associated with late-stage diagnosis. Public education about breast cancer and its early detection techniques is crucial to reduce mortality and improve the survival of patients. Besides, improving access to cancer screening services is useful to tackle the disease at its curable stages.

## Supporting information

S1 Checklist PRISMA 2020 checklist. (DOCX)

## References

[1] Park M, Kim D, Ko S, Kim A, Mo K, Yoon H: Breast cancer metastasis: Mechanisms and therapeutic implications. *International Journal of Molecular Sciences* 2022, 23(12):6806. doi: 10.3390/ijms23126806 35743249 PMC9224686

[2] Sung H, Ferlay J, Siegel RL, Laversanne M, Soerjomataram I, Jemal A, et al.: Global cancer statistics 2020: GLOBOCAN estimates of incidence and mortality worldwide for 36 cancers in 185 countries. *CA*: *a cancer journal for clinicians* 2021, 71(3):209–249. doi: 10.3322/caac.21660 33538338

[3] Wondimagegnehu A, Negash Bereded F, Assefa M, Teferra S, Zebrack B, Addissie A, et al.: Burden of cancer and utilization of local surgical treatment services in rural hospitals of Ethiopia: a retrospective assessment from 2014 to 2019. *The Oncologist* 2022, 27(11):e889–e898. doi: 10.1093/oncolo/oyac127 35791963 PMC9632304

[4] Ayele W, Führer A, Braun GA, Formazin F, Wienke A, Taylor L, et al.: Breast cancer morbidity and mortality in rural Ethiopia: data from 788 verbal autopsies. *BMC women’s health* 2022, 22(1):89. doi: 10.1186/s12905-022-01672-7 35331210 PMC8951700

[5] WHO: Global breast cancer initiative implementation framework: assessing, strengthening and scaling-up of services for the early detection and management of breast cancer: World Health Organization; 2023.

[6] Jedy-Agba E, McCormack V, Adebamowo C, Dos-Santos-Silva I: Stage at diagnosis of breast cancer in sub-Saharan Africa: a systematic review and meta-analysis. *The Lancet Global health* 2016, 4(12):e923–e935. doi: 10.1016/S2214-109X(16)30259-5 27855871 PMC5708541

[7] Shewarega B, Getachew S, Assefa N, Yesufe A, Trabizsch J, Dandena Y, et al.: Predictors of advanced-stage presentation among breast and cervical cancer patients in Ethiopia. *medRxiv* 2023:2023.2012. 2016.23300078.

[8] McGarvey N, Gitlin M, Fadli E, Chung KC: Increased healthcare costs by later stage cancer diagnosis. *BMC health services research* 2022, 22(1):1155. doi: 10.1186/s12913-022-08457-6 36096813 PMC9469540

[9] De Lemos LLP, Carvalho de Souza M, Pena Moreira D, Ribeiro Fernandes Almeida PH, Godman B, Verguet S, et al.: Stage at diagnosis and stage-specific survival of breast cancer in Latin America and the Caribbean: a systematic review and meta-analysis. *PloS one* 2019, 14(10):e0224012. doi: 10.1371/journal.pone.0224012 31618268 PMC6799865

[10] Tiruneh M, Tesfaw A, Tesfa D: Survival and predictors of mortality among breast cancer patients in Northwest Ethiopia: a retrospective cohort study. *Cancer Management and Research* 2021:9225–9234. doi: 10.2147/CMAR.S339988 34938122 PMC8687444

[11] Misganaw M, Zeleke H, Mulugeta H, Assefa B: Mortality rate and predictors among patients with breast cancer at a referral hospital in northwest Ethiopia: A retrospective follow-up study. *PloS one* 2023, 18(1):e0279656. doi: 10.1371/journal.pone.0279656 36701343 PMC9879427

[12] Riggio AI, Varley KE, Welm AL: The lingering mysteries of metastatic recurrence in breast cancer. *British journal of cancer* 2021, 124(1):13–26. doi: 10.1038/s41416-020-01161-4 33239679 PMC7782773

[13] Kiely BE: Does Stage at Diagnosis Affect Prognosis of Patients With Stage IV Breast, Lung, and Colorectal Cancers? *JNCI Cancer Spectrum* 2018, 2(2). doi: 10.1093/jncics/pky025 31360854 PMC6649771

[14] Sharma N, Purkayastha A: Factors Affecting Quality of Life in Breast Cancer Patients: A Descriptive and Cross-sectional Study with Review of Literature. *Journal of mid-life health* 2017, 8(2):75–83. doi: 10.4103/jmh.JMH_15_17 28706408 PMC5496284

[15] Darré T, Tchandikou L, Simgban P, Bombone M, Djiwa T, N’Timon B, Sama B, Ketevi A, Douaguibe B, N’Bortche BK et al: Factors associated with late diagnosis of breast cancer in women in Togo, Sub-Saharan Africa. *BMC women’s health* 2023, 23(1):106. doi: 10.1186/s12905-023-02257-8 36918873 PMC10012487

[16] Akhtar K, Akhtar K, Rahman MM: Use of Alternative Medicine Is Delaying Health-Seeking Behavior by Bangladeshi Breast Cancer Patients. *European journal of breast health* 2018, 14(3):166–172. doi: 10.5152/ejbh.2018.3929 30123883 PMC6092151

[17] Sobri FB, Bachtiar A, Panigoro SS, Ayuningtyas D, Gustada H, Yuswar PW, et al.: Factors Affecting Delayed Presentation and Diagnosis of Breast Cancer in Asian Developing Countries Women: A Systematic Review. *Asian Pacific journal of cancer prevention*: *APJCP* 2021, 22(10):3081–3092. doi: 10.31557/APJCP.2021.22.10.3081 34710982 PMC8858264

[18] Etissa EK, Assefa M, Ayele BT: Prognosis of colorectal cancer in Tikur Anbessa Specialized Hospital, the only oncology center in Ethiopia. *PloS one* 2021, 16(2):e0246424. doi: 10.1371/journal.pone.0246424 33529268 PMC7853488

[19] Gebremariam A, Addissie A, Worku A, Dereje N, Assefa M, Kantelhardt EJ, et al.: Association of Delay in Breast Cancer Diagnosis With Survival in Addis Ababa, Ethiopia: A Prospective Cohort Study. *JCO Global Oncology* 2023, 9:e2300148. doi: 10.1200/GO.23.00148 37992269 PMC10681531

[20] Tesfaw A, Tiruneh M, Tamire T, Yosef T: Factors associated with advanced-stage diagnosis of breast cancer in north-west Ethiopia: a cross-sectional study. *ecancermedicalscience* 2021, 15. doi: 10.3332/ecancer.2021.1214 33912239 PMC8057775

[21] Tesfaw A, Getachew S, Addissie A, Jemal A, Wienke A, Taylor L, et al.: Late-stage diagnosis and associated factors among breast cancer patients in south and southwest ethiopia: a multicenter study. *Clinical breast cancer* 2021, 21(1):e112–e119. doi: 10.1016/j.clbc.2020.08.011 33536135

[22] Hassen AM, Hussien FM, Asfaw ZA, Assen HE: Factors associated with delay in breast cancer presentation at the only oncology center in North East Ethiopia: a cross-sectional study. *Journal of Multidisciplinary Healthcare* 2021:681–694. doi: 10.2147/JMDH.S301337 33776446 PMC7989045

[23] Moher D, Liberati A, Tetzlaff J, Altman DG, Group* P: Preferred reporting items for systematic reviews and meta-analyses: the PRISMA statement. *Annals of internal medicine* 2009, 151(4):264–269. 19622511 10.7326/0003-4819-151-4-200908180-00135

[24] Munn Z, Moola S, Lisy K, Riitano D, Tufanaru C: Methodological guidance for systematic reviews of observational epidemiological studies reporting prevalence and cumulative incidence data. *JBI Evidence Implementation* 2015, 13(3):147–153. doi: 10.1097/XEB.0000000000000054 26317388

[25] Gebremariam A, Dereje N, Addissie A, Worku A, Assefa M, Abreha A, et al.: Factors associated with late-stage diagnosis of breast cancer among women in Addis Ababa, Ethiopia. *Breast Cancer Research and Treatment* 2021, 185:117–124. doi: 10.1007/s10549-020-05919-5 32948993

[26] Cserni G, Chmielik E, Cserni B, Tot T: The new TNM-based staging of breast cancer. *Virchows Archiv* 2018, 472:697–703. doi: 10.1007/s00428-018-2301-9 29380126

[27] Muhammed JA, Kroeber ES, Deribe B, Unverzagt S, Taylor L, Aynalem A, et al.: Prevalence and factors associated with delay in presentation of breast cancer patients in Ethiopia: A Cross-Sectional Institution-Based Study. *medRxiv* 2022:2022.2011. 2001.22281792.

[28] JBI: The Joanna Briggs Institute critical appraisal tools for use in JBI systematic reviews checklist for prevalence studies. North Adelaide: The Joanna Briggs Institute 2017.

[29] Munn Z, Tufanaru C, Aromataris E: JBI’s systematic reviews: data extraction and synthesis. *AJN The American Journal of Nursing* 2014, 114(7):49–54. doi: 10.1097/01.NAJ.0000451683.66447.89 25742352

[30] Huedo-Medina TB, Sánchez-Meca J, Marín-Martínez F, Botella J: Assessing heterogeneity in meta-analysis: Q statistic or I^2^ index? *Psychological methods* 2006, 11(2):193. 16784338 10.1037/1082-989X.11.2.193

[31] Egger M, Smith GD, Schneider M, Minder C: Bias in meta-analysis detected by a simple, graphical test. *BMJ (Clinical research ed)* 1997, 315(7109):629–634. doi: 10.1136/bmj.315.7109.629 9310563 PMC2127453

[32] Tesfaw LM, Teshale TA, Muluneh EK: Assessing the incidence, epidemiological description and associated risk factors of breast cancer in western Amhara, Ethiopia. *Breast Cancer Management* 2020, 9(3):BMT47.

[33] Kantelhardt EJ, Mathewos A, Aynalem A, Wondemagegnehu T, Jemal A, Vetter M, et al.: The prevalence of estrogen receptor-negative breast cancer in Ethiopia. *BMC cancer* 2014, 14:1–6. 25433805 10.1186/1471-2407-14-895 PMC4258259

[34] Yoseph M, Gebresadik A, Alemayehu A: Late Diagnosis of Breast Cancer and Associated Factors Among Women Attending Hawassa University Comprehensive and Specialized Hospital Southern Ethiopia. 2021.

[35] Teshome B, Trabitzsch J, Afework T, Addissie A, Kaba M, Kantelhardt EJ, et al.: Perceived barriers to timely treatment initiation and social support status among women with breast cancer in Ethiopia. *PloS one* 2021, 16(9):e0257163. doi: 10.1371/journal.pone.0257163 34516552 PMC8437283

[36] Legese B, Addissie A, Gizaw M, Tigneh W, Yilma T: Information needs of breast cancer patients attending care at Tikur Anbessa specialized hospital: A descriptive study. *Cancer Management and Research* 2021:277–286. doi: 10.2147/CMAR.S264526 33469370 PMC7812026

[37] Gebretsadik A, Bogale N, Negera DG: Epidemiological trends of breast cancer in southern Ethiopia: a seven-year retrospective review. *Cancer Control* 2021, 28:10732748211055262. 10.1177/10732748211055262 PMC8728771 34931549

[38] Koboto DD, Deribe B, Gebretsadik A, Ababi G, Bogale N, Geleta D, et al.: Quality of life among breast cancer patients attending Hawassa University comprehensive specialized hospital cancer treatment center. *Breast cancer*: *targets and therapy* 2020:87–95. doi: 10.2147/BCTT.S252030 32636670 PMC7335303

[39] Hassen AM, Taye G, Gizaw M, Hussien FM: Quality of life and associated factors among patients with breast cancer under chemotherapy at Tikur Anbessa specialized hospital, Addis Ababa, Ethiopia. *PloS one* 2019, 14(9):e0222629. doi: 10.1371/journal.pone.0222629 31539399 PMC6754151

[40] Dagne S, Abate SM, Tigeneh W, Engidawork E: Assessment of breast cancer treatment outcome at tikur anbessa specialized hospital adult oncology unit, Addis Ababa, Ethiopia. *European Journal of Oncology Pharmacy* 2019, 2(2):e13.

[41] Tessema Ersumo M, Girmaye Tamrat M, Bogale Solomon M, Tariku Gero M: BREAST CANCER IN A PRIVATE MEDICAL SERVICES CENTER: A 10-YEAR EXPERIENCE. *Ethiopian medical journal* 2018, 35:35–44.

[42] Tasfa Marine B, Mengistie DT: Application of parametric survival analysis to women patients with breast cancer at Jimma University Medical Center. *BMC cancer* 2023, 23(1):1223. doi: 10.1186/s12885-023-11685-6 38087229 PMC10714515

[43] Shita A, Yalew AW, Seife E, Afework T, Tesfaw A, Gufue ZH, et al.: Survival and predictors of breast cancer mortality in South Ethiopia: A retrospective cohort study. *PloS one* 2023, 18(3):e0282746. doi: 10.1371/journal.pone.0282746 36877683 PMC9987816

[44] Hagos BT, Gebrerufael GG: Time-to-death predictors on breast cancer patients in northern Ethiopia: a retrospective cross-sectional study. 2023.

[45] Feleke B, Tesfaw LM, Mitku AA: Survival analysis of women breast cancer patients in Northwest Amhara, Ethiopia. *Frontiers in Oncology* 2022, 12:1041245. doi: 10.3389/fonc.2022.1041245 36605442 PMC9808778

[46] Areri HA, Shibabaw W, Mulugeta T, Asmare Y, Yirga T: Survival status and predictors of mortality among Breast Cancer patients in Adult Oncology Unit at Black Lion Specialized Hospital, Addis Ababa, Ethiopia, 2018. *View at*: *Publisher Site* 2019.

[47] W/Michael Y: Factors associated to Late Diagnosis of Breast Cancer among women in Public and Private Hospital in Hawassa City, Southern Ethiopia. Addis Ababa University; 2018.

[48] Duggan C, Trapani D, Ilbawi AM, Fidarova E, Laversanne M, Curigliano G, et al.: National health system characteristics, breast cancer stage at diagnosis, and breast cancer mortality: a population-based analysis. *The Lancet Oncology* 2021, 22(11):1632–1642. doi: 10.1016/S1470-2045(21)00462-9 34653370

[49] Olayide A, Isiaka A, Ganiyu R, Samuel O, Halimat A, Julius O, et al.: Demographic pattern, tumor size and stage of breast cancer in africa: a meta-analysis. *Asian Pacific Journal of Cancer Care* 2021, 6(4):477–492.

[50] Bank TW: World Bank Country and Lending Groups Data. In.; 2024.

[51] Pearson JB: CARCINOMA OF THE BREAST IN NIGERIA. A REVIEW OF 100 PATIENTS. *British journal of cancer* 1963, 17(4):559–565. doi: 10.1038/bjc.1963.74 14117758 PMC2071219

[52] Galukande M, Wabinga H, Mirembe F, Karamagi C, Asea A: Difference in Risk Factors for Breast Cancer by ER Status in an Indigenous African Population. *ISRN oncology* 2013, 2013:463594. doi: 10.1155/2013/463594 23936673 PMC3708443

[53] Molassiotis A, Tyrovolas S, Giné-Vázquez I, Yeo W, Aapro M, Herrstedt J: Organized breast cancer screening not only reduces mortality from breast cancer but also significantly decreases disability-adjusted life years: analysis of the Global Burden of Disease Study and screening programme availability in 130 countries. *ESMO Open* 2021, 6(3):100111. doi: 10.1016/j.esmoop.2021.100111 33892452 PMC8085709

[54] Haileselassie W, Mulugeta T, Tigeneh W, Kaba M, Labisso WL: The Situation of Cancer Treatment in Ethiopia: Challenges and Opportunities. *Journal of cancer prevention* 2019, 24(1):33–42. doi: 10.15430/JCP.2019.24.1.33 30993093 PMC6453587

[55] Nguyen-Pham S, Leung J, McLaughlin D: Disparities in breast cancer stage at diagnosis in urban and rural adult women: a systematic review and meta-analysis. *Annals of Epidemiology* 2014, 24(3):228–235. doi: 10.1016/j.annepidem.2013.12.002 24462273

[56] Baade PD, Turrell G, Aitken JF: Geographic remoteness, area-level socio-economic disadvantage and advanced breast cancer: a cross-sectional, multilevel study. *Journal of epidemiology and community health* 2011, 65(11):1037–1043. doi: 10.1136/jech.2010.114777 21282144

[57] Israel E, Awoke N, Yakob T, Aynalem A, Talto A, Bezabih K: Determinants of breast self-examination practice among women attending pastoralist health facilities, Southern Ethiopia: a cross-sectional study. *BMC women’s health* 2023, 23(1):14. doi: 10.1186/s12905-023-02158-w 36627644 PMC9832667

[58] Udoh RH, Tahiru M, Ansu-Mensah M, Bawontuo V, Danquah FI, Kuupiel D: Women’s knowledge, attitude, and practice of breast self- examination in sub-Saharan Africa: a scoping review. *Archives of Public Health* 2020, 78(1):84. doi: 10.1186/s13690-020-00452-9 32974016 PMC7507650

[59] Getachew S, Tesfaw A, Kaba M, Wienke A, Taylor L, Kantelhardt EJ, et al.: Perceived barriers to early diagnosis of breast Cancer in south and southwestern Ethiopia: a qualitative study. *BMC women’s health* 2020, 20(1):38. doi: 10.1186/s12905-020-00909-7 32103774 PMC7045514

[60] Gangane N, Anshu, Manvatkar S, Ng N, Hurtig AK, San Sebastián M: Prevalence and Risk Factors for Patient Delay Among Women With Breast Cancer in Rural India. *Asia-Pacific journal of public health* 2016, 28(1):72–82. doi: 10.1177/1010539515620630 26658324

[61] BK M, Kaphle HP: Breast self-examination: Knowledge, practice and associated factors among 20 to 49 years aged women in Butwal sub-metropolitan, Rupandehi, Nepal. *PloS one* 2023, 18(6):e0286676. doi: 10.1371/journal.pone.0286676 37267248 PMC10237491

[62] Black E, Richmond R: Improving early detection of breast cancer in sub-Saharan Africa: why mammography may not be the way forward. *Globalization and health* 2019, 15:1–11. 30621753 10.1186/s12992-018-0446-6 PMC6325810

